# Supplementary material for: Mechanism for sound dissipation in a two-dimensional degenerate Fermi gas
Source: Sci Rep. 2024 May 11;14:10815. doi: 10.1038/s41598-024-61521-5 (PMC11088693; doi:10.1038/s41598-024-61521-5)
Supplement: Supplementary file 1 — Supplementary Information. [file 41598_2024_61521_MOESM1_ESM.pdf]

Suplementary materials for 'Mechanism for sound dissipation in a two-dimensional degenerate Fermi gas'

Krzysztof Gawryluk[1,*] **and Mirosław Brewczyk**[1]

[1]Wydział Fizyki, Uniwersytet w Białymstoku, ul. K. Ciołkowskiego 1L, PL-15245 Białystok, Poland
[*]k.gawryluk@uwb.edu.pl

ABSTRACT

Here we derive an expression for the interaction energy of a uniform balanced Fermi-Fermi system. The lowest-order constrained variational method (LOCV) is used. The result appears as an additional term in the equation of motion, see the main text.

1 LOCV approximation in two dimensions

The Hamiltonian of a uniform two-component two-dimensional (2D) degenerate Fermi gas is given by

$$H=-\frac{\hbar^2}{2m}\sum_{i=1}^{N_+}\nabla_i^2-\frac{\hbar^2}{2m}\sum_{j=1}^{N_-}\nabla_j^2+\sum_{i=1}^{N_+}\sum_{j=1}^{N_-}V_{FF}(\mathbf{x}_i-\mathbf{y}_j)\,, \tag{1}$$

where V_{FF} is a zero-range pseudopotential that lead to the scattering length in two dimensions, a_{2D}. The many-body ground state of H is assumed to be given in the form of the Jastrow-Slater variational wave function

$$|\Psi_{JS}\rangle=\prod_{i,j}f(\mathbf{x}_i-\mathbf{y}_j)\;|\Psi_S^+\rangle\,|\Psi_S^-\rangle\,, \tag{2}$$

where $|\Psi_S^{\pm}\rangle$ is the Slater determinant wave function of a component consisting of $N_{\pm}$ fermions and $f(\mathbf{r})$ is the Jastrow function describing the two-body correlations between interacting fermions. The pair correlation function, which is assumed to be spherically symmetric, is determined variationally by minimizing the average value of the energy $\langle\Psi_{JS}|H|\Psi_{JS}\rangle/\langle\Psi_{JS}|\Psi_{JS}\rangle$. Within the LOCV approximation the correlation function fulfills the Schrödinger equation

$$-\frac{\hbar^2}{m}\left(\frac{d^2f}{dr^2}+\frac{1}{r}\frac{df}{dr}\right)+V_{FF}(r)\,f(r)=\xi\,f(r) \tag{3}$$

in a space region defined by $r<d$. The healing length d, which is of the order of an average atomic separation, is determined self-consistently from the conditions $f(r>d)=1$ and $f'(r=d)=0$. For $r>d$, $f(r)$ tends to unity and the correlations disappear from (2). In the LOCV method the interaction energy E_{int}/N is approximated by

$$E_{int}/N=2\pi n\,\xi\int_0^d r\,|f(r)|^2\,dr \tag{4}$$

and d is chosen such that

$$2\pi n\,\xi\int_0^d r\,|f(r)|^2\,dr=1\,, \tag{5}$$

where n is the atomic density. Hence the interaction energy is $E_{int}/N=\xi$ (on average there are only correlated pairs). For the scattering state (the second lowest branch) $\xi=\hbar^2k^2/m>0$ and in the noninteracting case the solution of Eq. (3) is

$$f(r)\propto a(k)J_0(kr)+b(k)Y_0(kr)\,, \tag{6}$$

where $a(k)$ and $b(k)$ are coefficients set by the boundary conditions and $J_0(kr)$ and $Y_0(kr)$ are Bessel functions of the first and second kinds, respectively. The two-dimensional contact interactions can be introduced by imposing the Bethe-Peierls boundary conditions at $r = 0$

$$\left(r\frac{d}{dr} - \frac{1}{\ln(r/a_{2D})} \right) f(r) \underset{r\to 0}{\longrightarrow} 0 \,, \tag{7}$$

which gives[1]

$$f(r) \propto \left\{ J_0(kr) - \frac{\pi}{2[\gamma + \ln(k a_{2D}/2)]} Y_0(kr) \right\} \,, \tag{8}$$

where $\gamma \approx 0.577$ is the Euler's constant. Since $f(r = d) = 1$, the constant of proportionality equals

$$\left\{ J_0(kd) - \frac{\pi}{2[\gamma + \ln(k a_{2D}/2)]} Y_0(kd) \right\}^{-1} \,. \tag{9}$$

To determine the healing length (d) and the interaction energy per particle ($\sim k^2$), the constraint (5) and the condition $f'(r = d) = 0$ have to be used. The second condition yields

$$J_1(kd) = \frac{\pi}{2[\gamma + \ln(k a_{2D}/2)]} Y_1(kd) \,. \tag{10}$$

Set of nonlinear equations (5) and (10) can be solved numerically giving quantities (k, d) for each value of 2D interaction parameter $\eta = \ln(k_F a_{2D})$, where $k_F = \sqrt{4\pi n}$ is the Fermi momentum of a two-dimensional single-component Fermi gas of density n. Similar considerations can be repeated for the bound state (the lowest energy branch), just by making a replacement $k \to i\kappa$ (then $\xi = -\hbar^2\kappa^2/m < 0$).

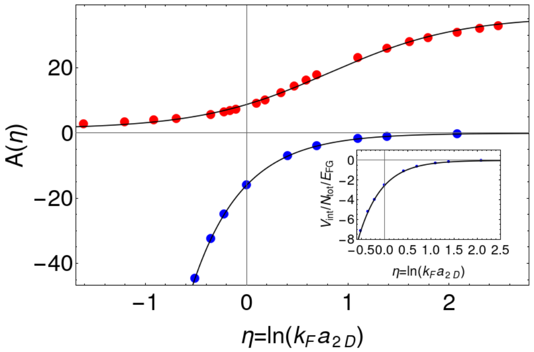

Figure 1. $A(\eta)$, where $\eta = \ln(k_F\, a_{2D})$, calculated within 2D LOCV approximation for two lowest energy branches. The solid black lines are just a guide to the eye. Inset shows the interaction energy per particle in units of the energy per particle of the noninteracting gas, $V_{int}/N_{tot}/E_{FG}$, where $E_{FG} = \pi(\hbar^2/m)n$. The data compare well with the results of the fixed-node diffusion Monte Carlo calculations of Ref.[2] (see Fig. 1).

Now, the interaction energy density of each uniform component, calculated within the LOCV approximation, is $E^{\pm}_{int}/V = \xi n_{\pm} = 4\pi(\hbar^2/m)\, n_{\pm} n_{\mp} (k/k_F^{\mp})^2$. Hence, the total interaction energy density is

$$E_{int}/V = (E^{+}_{int}/V + E^{-}_{int}/V)/2 = (\hbar^2/2m)\, n_+ n_- \,[A(\eta_+) + A(\eta_-)] \,, \tag{11}$$

where $A(\eta) = 4\pi(k/k_F)^2$. The function $A(\eta)$ is shown in Fig. 1 for two lowest energy branches. Applying the local density approximation the interaction energy functional of a two-component two-dimensional Fermi gas can be written as

$$V_{int} = \int \frac{\hbar^2}{2m} n_+(\mathbf{r})\, n_-(\mathbf{r})\, [A(\eta_+(\mathbf{r})) + A(\eta_-(\mathbf{r}))]\, d\mathbf{r} \,. \tag{12}$$

Then the contribution to equations of motion, Eqs. (4) main text, due to interactions (the chemical potential μ_{int}) becomes

$$\frac{\delta V_{int}}{\delta n_{\pm}} = \frac{\hbar^2}{2m} n_{\mp} \left[A(\eta_+) + A(\eta_-) + \frac{1}{2}\frac{dA(\eta)}{d\eta}\Big|_{\eta_{\pm}} \right] \tag{13}$$

and the cutoff energy for a spin-balanced mixture consisted of $\langle N_{\pm} \rangle = 1500$ atoms at the temperature $T/T_F = 0.2$ as a function of 2D interaction parameter is plotted in Fig. 2. The inset depicts the chemical potential μ_{int}.

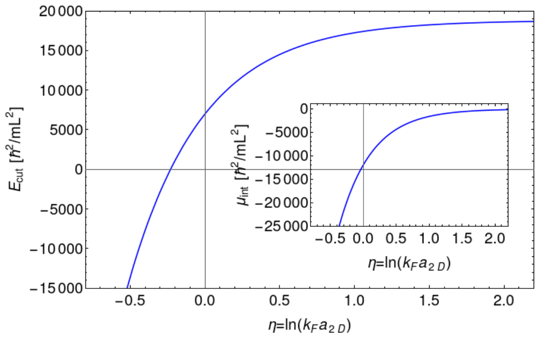

Figure 2. Cutoff energy as a function of $\eta = \ln(k_F\, a_{2D})$ for the mixture consisted of $\langle N_{\pm} \rangle = 1500$ atoms at the temperature $T/T_F = 0.2$. Inset shows the chemical potential μ_{int}.

References

1. T.M. Whitehead, L.M. Schonenberg, N. Kongsuwan, R.J. Needs, and G.J. Conduit, Phys. Rev. A **93**, 042702 (2016).
2. G. Bertaina and S. Giorgini, Phys. Rev. Lett. **106**, 110403 (2011).
